# Serum Interleukin-4 and Total Immunoglobulin E in Nonatopic Alopecia Areata Patients and HLA-DRB1 Typing

**DOI:** 10.1155/2010/503587

**Published:** 2010-06-30

**Authors:** Enas A. S. Attia, Dina El Shennawy, Ashraf Sefin

**Affiliations:** ^1^Department of Dermatology, Venereology, and Andrology, Faculty of Medicine, Ain Shams University, Cairo 11566, Egypt; ^2^Department of Clinical Pathology, Faculty of Medicine, Ain Shams University, Cairo 11566, Egypt; ^3^Department of Public Health, Faculty of Medicine, Ain Shams University, Cairo 11566, Egypt

## Abstract

*Background.* Interleukin-4 (IL-4), a Th_2_ cytokine, can stimulate immunoglobulin E (IgE) transcription. No previous studies evaluated the genetic mechanisms in nonatopic AA patients with elevated serum IgE. *Objective.* To compare serum IL-4 and total IgE levels between Egyptian nonatopic AA patients and healthy subjects and to investigate a possible relation to HLA-DRB1 alleles. *Results.* Serum IL-4 and total IgE were measured by ELISA in 40 controls and 54 nonatopic AA patients. Patients' HLA-DRB1 typing by sequence specific oligonucleotide probe technique was compared to normal Egyptian population. We found significantly elevated serum IL-4 and total IgE in AA patients (particularly alopecia universalis, AU, and chronic patients) (*P* < .01). HLA-DRB1*11 is a general susceptibility/chronicity allele. DRB1*13 is a protective allele. DRB1*01 and DRB1*07 are linked to chronicity. Localized AA showed decreased DRB1*03 and DRB1*07. Extensive forms showed increased DRB1*08 and decreased DRB1*04. Elevated IL4 and IgE were observed in patients with DRB1*07 and DRB1*11 not DRB1*04. *Conclusion.* Serum IL-4 and IgE are elevated in nonatopic AA patients, particularly AU and chronic disease. Relevant susceptibility, chronicity, and severity HLADRB1 alleles may have a role in determining type, magnitude, and duration of immune response in AA favouring increased IL4 and IgE.

## 1. Introduction

Alopecia areata (AA) is an unpredictable, usually patchy, nonscarring hair loss condition with any hair-bearing area may be affected [1]. Papadopoulos et al. classified AA according to the extent of hair loss into: localized or patchy AA (LAA), alopecia totalis (AT), with complete loss scalp hair, and alopecia universalis (AU) with total body hair loss [2]. Of the numerous pathogenic processes which have been proposed to underlie the etiology of AA, immunological, environmental, psychological, and genetic factors are the most powerful explanations [3].

Although increased levels of T helper1 (Th_1_) cytokines (interferon-*γ*, IFN-*γ* and interleukin-2, IL-2) in lesional AA skin have been reported [4], Th_2_ immune response is also incriminated in the pathogenesis of AA [5, 6]. Cytokines derived from Th_2_ cells, for example, IL-4 and IL-13 can stimulate the transcription of IgE in B-cells through Ig constant region genes [7, 8]. Unfortunately, the mechanism by which IgE might interact in the pathogenesis of AA is unknown. One possible explanation is supported by the immune status of AA [5]. However, Different studies have measured IgE in AA patients with controversial results [9, 10]. Another possibility to explain the elevation of total serum IgE in AA patients is the genetic mechanisms of AA, which seems to be polygenic, where several genes such as IL-4 gene [8], and the gene for *β* subunit of type 1 IgE receptor (Fc*ε* RI*β*) [6], play a role in determining the disease susceptibility, and the associations of AA with atopic disease [11]. However, to our knowledge, no previous studies were done to evaluate the genetic mechanisms in nonatopic AA patients with elevated serum IgE levels.

As is the case with many other autoimmune diseases, there is an association between AA and HLA. The most significant associations between AA and HLA loci vary with different populations as HLA antigens are population specific [12]. 

Therefore, the aim of this work was to compare serum levels of IL-4 and total IgE between Egyptian patients with different clinical forms of AA without atopic background and healthy subjects, and to investigate the relation between these levels to the HLA-DRB1 profile, the most frequent HLA-DR alleles among Egyptians.

## 2. Subjects and Methods

### 2.1. Subjects

After giving informed consent, 54 patients with AA were included in the present study, collected from the Dermatology Outpatient Clinic, Ain Shams University Hospital during the period from July 2008 till June 2009. They were 36 males and 18 females, and their age ranged from 20 to 65 years. The control group consisted of 40 age- and sex-matched generally healthy subjects, 26 males and 14 females, with no scalp lesions in their personal history or on clinical examination. All subjects of the study (patients and controls) were subjected to: full history taking and dermatological examination including: search for possible associations with AA such as atopic dermatitis and vitiligo and clinical assessment of AA lesions including: number of the lesions, site and size of the lesions, and classification according to clinical severity into: LAA, AT, and AU. All subjects of the study were subjected to: stool analysis to exclude parasitic infestation, complete blood count (CBC) to determine the number of eosinophils, and skin prick test to a panel of allergens. Patients using topical, intralesional, or systemic agents as steroids or immunosuppressives likely to cause regrowth in AA within the past month, patients subjected to sessions of PUVA, for at least 6 months before this study, and those having other types of illness such as atopic diseases and parasitic infestation or those who showed positive response to skin prick test, that could affect the outcome of the study, were excluded.

### 2.2. Methods

Both patients and controls were subjected to determination of serum IL-4 and total IgE levels using enzyme-linked immunosorbent assay (ELISA) technique (AviBion Human IL4 ELISA Kit. FINLAND for IL4 and Monobind Inc. USA for IgE). The assay was performed in a blind fashion on coded samples by an investigator who was not informed of the subject's clinical status, after the collection of all samples had been completed. In healthy, non allergic adults, reference range of IgE was up to 90 IU/ml.

HLA-DRB1 typing was done for patients by sequence specific oligonucleotide probe (SSOP) technique. Five ml blood on sterile EDTA tubes were collected from each subject, and DNA extractions were done using GIFXTM genomic blood DNA purification kit (Amersham Biosciences Inc., USA). Typing was done using Dynal RELITM SSO HLA-DRB typing kit (Dynal Biotech Ltd.,UK). Interpretation of the results was done using the Dynal RELITM SSO pattern matching program [13]. To compare their results with the normal population, control samples results were taken from the study population by Khalil et al. [14]. Their population included healthy unrelated Egyptian individuals (age range 23–38 years) attending at the immunogenetics and transplantation lab of Ain Shams University Specialized Hospital as kidney donors for their relatives.

Data analysis was done using SPSS software package (V. 16, Echosoft Corp., USA). Qualitative data were analyzed using Chi-square test while quantitative data were analyzed by Kruskal-Wallis and Mann-Whitney test. Pearson correlation coefficient was done for IgE and IL-4. The probability of error at 0.05 was considered significant, while at 0.01 is highly significant.

## 3. Results

The study included 54 patients with AA; among them, there were 18 patients (33.3%) with LAA, 18 (33.3%) with AT, and 18 (33.3%) with AU. The duration of AA ranged from 2 weeks to 9 years with a mean of 18.4 months. Forty-one patients (75.9%) had duration of disease of less than 1 year, and 13 patients (24.1%) their disease lasted more than 1 year. There were 4 patients (7.4%) with positive family history of AA. Ten patients (18.5%) showed a relation between the onset of their disease and a preceding period of stress, while 44 (81.5%) stated no relation to stress.

**Figure 1:**
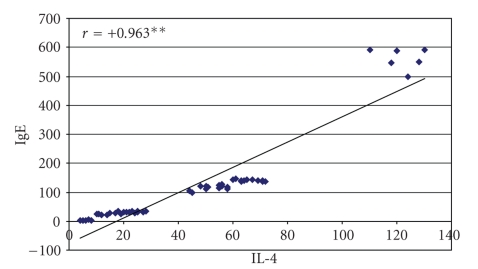
Correlation between serum IL-4 and IgE among the studied patients.

Twenty-six (48.3%) of our patients had elevated IgE. was statistically highly significant increase in both IgE and IL-4 levels in patients compared to controls was observed (median of 111 versus 21.1 IU/ml and 49 versus 12 pg/ml, resp.) (Table 1). Table 2 showed that there is highly significant difference in IgE and IL4 levels among different types of Alopecia (both were statistically significantly elevated in AU patients, compared to both AT and LAA). By doing Spearman correlation test between IgE and IL4 among patients, it was clear that there is a statistically significant positive correlation (elevated IgE is associated with increased IL4 level) (*r* = .963, *P* < .01) (Figure 1). The data presented in Table 3 indicated that there is a statistically highly significant increase in both IgE and IL4 levels in chronic patients with disease duration more than 1 year, but not with less than 1 year, in comparison to controls.

Concerning the frequencies of selected HLA-DRB1 alleles among patients and normal Egyptians, a statistically significant increase was elicited in patients regarding HLA-DRB1*11. In contrast, HLA-DRB1*13 was significantly decreased among patients. LAA patients showed a statistically significant decrease regarding HLA-DRB1*03 and HLA-DRB1*07, compared to controls. Comparing AT/AU patients and normal controls, a statistically significantly increased HLA-DRB1*08 and decreased HLA-DRB1*04 was found in patients (Table 4). In addition, AT/AU patients showed significantly decreased HLA-DRB1*04 compared to LAA (*P* = .003). Comparing patients with a disease duration of less and more than 1 year, both of the general susceptibility allele; HLA-DRB1*11, and HLA-DRB1*01 were linked to chronicity. In addition, HLA-DRB1*03 was found to protect against chronicity, while HLA-DRB1*07 predisposed to prolonged disease duration (Table 5). 

Using Chi square test, comparison of the frequencies of selected HLA-DRB1 alleles in alopecia of less and more than 1 year duration, in relation to IL-4/IgE levels, revealed that IL-4/IgE levels were statistically highly significantly elevated among the HLADRB1*11 positive chronic patients (having general AA susceptibility allele) and among the HLADRB1*07 positive chronic patients (harboring chronicity allele) (Table 6). For HLADRB*04, all patients had low IL-4/IgE levels; 17 of them had a disease duration of less than 1 year, while only one was a chronic case. Thus, there are lowered levels of IL-4 and IgE in patients with HLA-DR*04 which was absent in AT/AU (antiseverity allele). In other words, patients susceptible to AA, particularly chronic and extensive disease, are also susceptible to increased IL-4 and IgE levels. Other relevant alleles showed equivocal frequencies with respect to IL-4/IgE levels.

## 4. Discussion

We investigated serum IL-4 and total IgE levels in AA patients with exclusion of those with possible atopic disease to evaluate the role of immune signals for IgE induction, in AA patients, irrespective of atopic immune mechanisms. We demonstrated that 48.3% of AA patients had elevated total serum IgE, and that the mean serum levels of IL-4 and total serum IgE were significantly elevated in patients with AA in comparison to normal controls. The association of serum IgE levels and AA had been previously investigated with varying results. O'Loughlin et al. analyzed serum IgE of 497 patients with various dermatological diseases including AA, in comparison to 95 normal controls. They found elevated total serum IgE in 30% of AA patients [12]. Przybilla et al. found elevated total IgE in 19.7% of AA patients [9]. Kasumagić-Halilovíc and Prohić, also found elevated total IgE in 22 of 60 (37%) of AA patients [10]. However, in contrast to these results, total serum IgE levels were not elevated in AA patients in other previous studies [11, 15]. The discrepancy between our results and the results of some previous studies could be attributed to the difference in inclusion and exclusion criteria, the difference in number of patients involved, and to the different environmental factors which may influence IgE level. 

In addition to B-cell antigen receptor stimulation, Th_2_ cytokines can switch on IgE production in AA [7, 8]. Teraki et al. found that serum levels of IL-1*α* and IL-4 were significantly elevated in patients with the LAA, while serum levels of IFN-*γ* and IL-2 were significantly elevated in patients with extensive forms [5]. The same was suggested by Katagiri et al., who found that the levels of IFN-*γ* tended to increase while the levels of IL-4 tended to decrease in severe cases of alopecia [6]. In contrast, we found that total serum IgE and IL-4 were elevated more in AU than in other forms. This could be explained by the fact that previous studies did not exclude patients with atopy and did not relate cytokine profile to disease chronicity. Thus, the speculation of different immune responses in LAA from the extensive forms, being regulated by Th_2_ cytokines and Th_1_ cytokines, respectively, needs further studies.

Our study provided a correlation between the onset of AA and the level of IL-4 and IgE, since the longer the disease duration was, the higher the levels of IL-4 and IgE were present. Previous mouse and human data suggested that the initiation phase of AA is a heavily Th_1_-biased immune response, while the maintenance of destruction of hair follicles by cytotoxic cells is due to a shift from a Th_1_ response to a more chronic Th_2_ immune profile [16]. Thus, AA is a cell-mediated autoimmune disease with late, possibly secondary, humoral responses.

Regarding HLA-DRB1 profile, our results suggest that there is positive association between AA among Egyptian patients and HLA-DRB1*11. On the other hand, HLA-DRB1*13 is a protective allele. Besides, patients with HLA-DRB1*07 were likely to develop LAA, while patients with HLA-DRB1*03 were protected against it. Patients having HLA-DRB1*04 are less likely to develop the extensive forms, while having HLA-DRB1*08 make them susceptible to extensive disease. In agreement with some of our results, Broniarczyk-Dyla et al. identified DRB1*03 as a protective allele against AA [17]. Akar et al. found that the frequency of DRB1*03 was decreased in all Turkish patients group, while DRB1*04 was increased in patients with 25%–75% scalp hair loss [18]. Morling et al. reported significant association between AA and DRB1*1104 allele [19]. Colombe et al. also identified increased DRB1*1104 in all American AA patients groups, but they found DRB1*0401 (DR4) to be significantly increased uniquely in AT/AU patients [20, 21]. Barahmani et al. found DRB1*1104 to be strongly associated and DRB1*0301 to be negatively associated with AT/AU [22]. A significant increase of DR4 was also reported in Caucasian British AA patients by Zhang et al. [23]. Association with DR4 has also been found by several groups [24–27].

We noticed that the general susceptibility allele; HLA-DRB1*11, and DRB1*01 and DRB1*07 were linked to chronicity, while HLA-DRB1*03 was found to protect against disease chronicity. In contrast, Akar et al. found no significant difference in the frequency of HLA alleles between early and late onset disease patients [18], while Orecchia et al. did find a correlation with DR5 (DR11) in Italian patients [28].

HLA-DRB1*11 and HLA-DRB1*07 (general AA susceptibility and chronicity alleles) were associated with elevated levels of serum IL4 and IgE in chronic patients. However, with HLA-DRB1*4 (antiseverity allele) there were lowered levels of both. This means that the relevant HLA-DRB1 alleles with respect to susceptibility, chronicity, and severity of AA may have an important role in determination of immune response, favouring increased IL4 and IgE. In other words, patients susceptible to AA in general, and to the chronic and severe forms in particular, are also susceptible to increased IgE and IL4 levels.

## 5. Conclusion

In conclusion, serum IL-4 and IgE are significantly elevated in AA patients, particularly AU, irrespective of the presence of atopy. Our study also provided a correlation between the onset of AA and serum levels of IgE and IL-4. This finding suggests a shift from a Th_1_ response in early AA to a more chronic Th_2_ immune profile, with secondary B-cell stimulation and possible IgE class switching. Elevated IgE and IL-4 could be related to the HLA profile of patients, which controls the type, magnitude, and duration of immune response, contributing to disease susceptibility, severity, and chronicity.

## Figures and Tables

**Table 1:** Comparison of IgE and IL-4 levels between cases and controls using Mann-Whitney test.

	Cases *n* = 54	Controls *n* = 40	*Z* Mann Whitney	*P* value
IgE: Median	111	21.1	4.470	<.001**
(IU/ml) SEM	15.58	6.72		

IL-4: Median	49	12	6.366	<.001**
(Pg/ml) SEM	3.3	1.03		

SEM: Standard error of mean.

**statistically highly significant difference.

**Table 2:** Comparison between IgE and IL4 levels in different clinical forms of AA using Kruskal-Wallis test.

Type of alopecia		IgE (IU/ml)	IL4 (Pg/ml)
AU	Median	141.0	66.0
	SEM	38.68	6.77

AT	Median	111.0	49.0
	SEM	6.81	2.62

LAA	Median	25.0	13.0
	SEM	10.14	4.5

Kruskal-Wallis		28.06	28.94

*P* value		<.001**	<.001**

AT: alopecia totalis; AU: alopecia universalis; LAA: localized alopecia areata.

SEM: Standard error of mean.

**statistically highly significant difference.

**Table 3:** Comparison between IgE and IL4 levels in patients with disease duration less and more than 1 year and controls using Kruskal-Wallis test.

Duration class		IgE (IU/ml)	IL4 (Pg/ml)
Less than 1 year	Median	34.0	28.0
	SEM	9.06	3.02

More than 1 year	Median	112.0	51.0
	SEM	19.95	4.19

Control	Median	21.1	12.0
	SEM	6.72	1.03

Kruskal-Wallis		20.135	40.539

*P* value		<.001**	<.001**

SEM: Standard error of mean.

**statistically highly significant difference.

**Table 4:** Frequencies of selected HLA-DRB1 alleles in alopecia patients compared to controls using Chi square test.

DRB1 allele	Controls (*n* = 580)	Overall alopecia (*n* = 54)	*P* value	LAA (*n* = 18)	*P* value	AT/AU (*n* = 36)	*P *value
HLA-DRB1*01	5%	5.6%	.998	0%	.083	5.6%	.954
HLA-DRB1*03	19%	22.2%	.992	5.5%	.000*	16.6%	.515
HLA-DRB1*04	16%	16.7%	1.0	11.1%	.223	5.6%	.006**
HLA-DRB1*07	6%	11.1%	.31	0%	.04*	11.1%	.088
HLA-DRB1*08	2%	5.6%	.28	0%	.911	5.6%	.002**
HLA-DRB1*11	12%	22.2%	.048*	11.1%	.735	11.1%	.735
HLA-DRB1*13	16%	5.6%	.021*	0%	.00**	5.6%	.003**
HLA-DRB1*15	7%	11.1%	.459	5.5%	.658	5.6%	.658

AT: alopecia totalis; AU: alopecia universalis; LAA: localized alopecia ateata.

*statistically significant difference.

**statistically highly significant difference.

**Table 5:** Frequencies of selected HLA-DRB1 alleles in Alopecia of less than 1 year duration compared to alopecia with a duration of more than 1 year using Chi square test.

DRB1 Allele	Alopecia less than 1 year (*n* = 41)	Alopecia more than 1 year (*n* = 13)	*P* value
HLA-DRB1*01	0%	23.1%	<.001**
HLA-DRB1*03	29.3%	0%	.014*
HLA-DRB1*04	20.7%	3.8%	.087
HLA-DRB1*07	6.1%	26.9%	.010**
HLA-DRB1*08	7.3%	0%	.866
HLA-DRB1*11	14.6%	46.2%	.002**
HLA-DRB1*13	7.3%	0%	.866
HLA-DRB1*15	14.6%	0%	.26

*statistically significant difference.

**statistically highly significant difference.

**Table 6:** Comparison of the frequencies of selected HLA-DRB1 alleles in Alopecia of less and more than 1 year duration, in relation to IgE/IL-4 level using Chi square test.

Allele	Duration	High IgE/IL-4 (*n* & %)	Low IgE/IL-4 (*n* & %)	*x* ^2^	*P* value
HLA-DRB1*07	Less than 1 year	0 (0%)	5 (83%)	8.571	.003**
	More than 1 year	6 (100%)	1 (16.7%)		

HLA-DRB1*11	Less than 1 year	0 (0%)	12 (66.7%)	8.00	.005**
	More than 1 year	6 (100%)	6 (33.3%)		

**statistically highly significant difference.
